# Early treatment with hydroxychloroquine prevents the development of endothelial dysfunction in a murine model of systemic lupus erythematosus

**DOI:** 10.1186/s13075-015-0790-3

**Published:** 2015-10-06

**Authors:** Agostino Virdis, Chiara Tani, Emiliano Duranti, Sabrina Vagnani, Linda Carli, Anja A. Kühl, Anna Solini, Chiara Baldini, Rosaria Talarico, Stefano Bombardieri, Stefano Taddei, Marta Mosca

**Affiliations:** Department of Clinical and Experimental Medicine, University of Pisa, Pisa, Italy; Rheumatology Unit, Department of Clinical and Experimental Medicine, University of Pisa, Via Roma, 67, 56100 Pisa, Italy; GenOMeC PhD, University of Siena, Siena, Italy; Medical Clinic I for Gastroenterology, Infectiology and Rheumatology, Campus Benjamin Franklin, Charité - Universitätsmedizin Berlin, Hindenburgdamm 30, D-12203 Berlin, Germany

## Abstract

**Introduction:**

Accelerated atherosclerosis is one of the major causes of morbidity in patients with systemic lupus erythematosus (SLE). Endothelial dysfunction (ED) is considered an early marker of atherosclerosis. It is a reversible alteration, thus representing an attractive target for prevention strategies against cardiovascular disease. Studies have shown that ED occurs in patients with SLE even in the absence of severe, active disease. Hydroxychloroquine (HCQ) is widely used in SLE to control disease activity, but its use is also associated with an improvement in long-term prognosis. Beyond the beneficial effect in well-established disease, our hypothesis is that treatment with HCQ might have a beneficial impact on ED prevention in SLE. The aim of this study was to assess the impact of early treatment with HCQ on ED in a murine model of SLE.

**Methods:**

Twelve-week-old NZB/W F1 (NZ) and C57BL/6 J mice (controls) were allocated to receive HCQ or vehicle for 6, 12, or 18 weeks. Proteinuria and anti–double-stranded DNA autoantibodies were determined. ED was assessed in mesenteric arteries (pressurized myography). Nitric oxide (NO) availability and reactive oxygen species (ROS) production were evaluated. Vascular ROS production was measured with dihydroethidium (DHE) fluorescent dye.

**Results:**

Starting from 18 weeks of age, NZ mice showed a progressive reduction in NO availability, which was normalized by ascorbic acid and apocynin in the up to 24-week-old group, and partly ameliorated in older animals. HCQ administration normalized the NO availability in the up to 24-week-old group, with a partial amelioration in the 30-week-old group. DHE analysis revealed a progressive increment of vascular ROS generation among NZ groups, which was prevented by apocynin. Similarly, in the NZ HCQ-treated group, vascular ROS production was abrogated.

**Conclusions:**

The ED that characterizes this mouse model of SLE is caused by the nicotinamide adenine dinucleotide phosphate oxidase–driven ROS excess. Very early treatment with HCQ is able to exert vascular protection via an antioxidant effect.

**Electronic supplementary material:**

The online version of this article (doi:10.1186/s13075-015-0790-3) contains supplementary material, which is available to authorized users.

## Introduction

In recent years, the survival of patients with systemic lupus erythematosus (SLE) has increased substantially. Reasons for the improved prognosis include earlier diagnosis, enhanced recognition of milder forms, and mindful use of treatments such as glucocorticoids and immunosuppressive agents. However, because of prolonged life expectancy, patients with SLE are now exposed to an increased risk of morbidity related to the sequelae of disease activity, side effects of medications, and comorbid conditions, such as premature cardiovascular (CV) morbidity [1, 2].

Accelerated atherosclerosis is one of the major causes of CV morbidity and mortality in patients with SLE. Of note, over the past 3 decades, mortality has been significantly reduced for all causes among patients with SLE, with the exception of CV disease. For these reasons, the prevention of long-term CV complications in patients with SLE, while still an unsolved issue, represents a particularly attractive target for intervention. Beyond the concomitance with traditional CV risk factors, including obesity, hypertension, dyslipidemia, and diabetes mellitus, SLE is considered per se responsible for direct detrimental effects on the vasculature, which makes SLE itself a potent, independent CV risk factor [3, 4].

Endothelial dysfunction (ED) is characterized by impaired nitric oxide (NO) availability and a concomitant increased reactive oxygen species (ROS) generation [5]. Such a systemic condition is shared by the majority of CV risk factors, is recognized as an early and major promoter for atherosclerosis and thrombosis, and is independently related to CV events [5]. Interestingly, SLE and atherosclerosis share mechanisms such as vascular inflammation, ROS excess, immune complexes, and complement activation, which are all able to elicit ED. Of note, ED is a reversible alteration, thus representing a potentially attractive target for preventive intervention against CV disease.

Hydroxychloroquine (HCQ) is an antimalarial compound with immunomodulatory properties widely used in SLE owing to its multiple beneficial effects, including control of disease activity, reduction of damage accrual, improvement of survival, and a relatively safe profile [6–10]. In recent years, HCQ therapy has been associated with an improvement of CV prognosis in SLE. A positive effect on traditional CV risk factors and on thrombotic risk has been described in SLE [11–14]; moreover, a beneficial effect on vascular reactivity indices has also been reported [15, 16].

However, it is still unclear whether the protective CV effect of antimalarials is the indirect result of better control of disease activity or, alternatively, a direct effect of HCQ on the endothelial microenvironment. The availability of lupus murine models offers a great opportunity to study this relationship in a “simplified” setting to improve understanding of the role of the multiple contributing factors.

Recently, Gómez-Guzmán et al. [17] have shown that short-term treatment with HCQ started at a late disease stage is able to reverse ED at the level of large arteries in a murine model of SLE, an effect mediated by a reduction of the nicotinamide adenine dinucleotide phosphate [NAD(P)H] oxidase activity, which is a major ROS source. Beyond the beneficial effect of the antimalarial in conditions of well-established disease, early HCQ use has been also associated with delayed SLE onset in humans [18].

We hypothesized that treatment with HCQ before the onset of the full-blown disease might delay or even prevent SLE-associated ED. The present study was designed to assess the effect of HCQ on endothelial function in a murine model of SLE and its underlying mechanism.

## Methods

### Animal treatment

All experiments were carried out in accordance with European Union Council Directive 2010/63/EU. Female NZB/W F1 (NZ) mice and C57BL/6 J mice (controls; Ctrl) were obtained from Harlan Laboratories (Correzzana, Italy) and housed in a specific pathogen-free barrier facility. Starting from 12 weeks of age, the animals were randomly allocated to receive HCQ (3 mg/kg/day) or vehicle (sham) in the drinking water. The animals were divided into four groups: untreated control (Ctrl; n = 15), NZB/W F1 mice with untreated SLE (NZ; n = 30), control treated with HCQ (Ctrl-HCQ; n = 30), and NZB/W F1 mice with SLE treated with HCQ (NZ-HCQ, N = 30). The mice were treated for three different periods (6, 12, or 18 weeks) (Table 1). At the end of treatment, they were killed while under avertin-induced anesthesia. The protocol was approved by the local University of Pisa ethics committee on use and care of animals.

**Table 1:** 24-h proteinuria

Age, wk	Group	Mean	SD	Group	Mean	SD	Group	Mean	SD
8–12	NZ	0.30	0.1	NZ_HCQ_	0.22	0.02	Ctrl	1.35	1.23
18	NZ	0.56	0.44	NZ_HCQ_	0.18	0.40	Ctrl	0.46	0.42
20	NZ	3.5^a^	2.7	NZ_HCQ_	0.43	0.15	Ctrl	0.78	0.6
24	NZ	4.06	2.8	NZ_HCQ_	0.23	0.10	Ctrl	0.34	0.40
30	NZ	4.9	2.2	NZ_HCQ_	0.35	0.34	Ctrl	0.27	0.07
36	NZ	12.5	3.5	NZ_HCQ_	12.18^b^	6.3	Ctrl	1.083	0.50

*Ctrl* untreated control, *NZ* female NZB/W F1, *NZ*_*HCQ*_ NZB/W F1 mice with systemic lupus erythematosus treated with hydroxychloroquine, *SD* standard deviation

Data are presented in milligrams per 24 h

^a^ *P* < 0.05 at 20 weeks vs. 18 weeks

^b^ *P* < 0.05 at 36 weeks vs. 30 weeks

### Serum and urinary assays

At the time the mice were killed, blood samples were collected and immediately placed in heparinized tubes. Serum was separated by centrifugation and stored at −70 °C. Serum titers of anti–double-stranded DNA (anti-dsDNA) autoantibodies were analyzed with a commercial enzyme-linked immunosorbent assay kit (Alpha Diagnostic International, San Antonio, TX, USA), 24-h urinary protein excretion was collected in metabolic cages and measured by dipstick analysis (Bayer, Leverkusen, Germany). For both serum and urine assays, the person who performed the measurements was blinded to the age and treatment allocation of the mice.

### Histopathology

The kidneys were excised, fixed overnight in 4 % formalin, and embedded in paraffin. Sections (1–2 μm) were cut, dewaxed, and stained with hematoxylin and eosin (H&E) and periodic acid–Schiff (PAS). H&E- and PAS-stained sections were evaluated for nephritis according to a modified score described by Tao et al. as follows [19]:

- *Glomerulonephritis*: 1, focal and mild hypercellularity; 2, multifocal and moderate hypercellularity with capillary dilation and mild hyalinosis; 3, diffuse hypercellularity (>50 % of the tuft) and capillary aneurysm; 4, extensive sclerosis and/or crescents (more than three cell layers), tuft obliteration, collapse
- Interstitial nephritis: 1, small foci of leukocyte infiltration; 2, mild inflammation of individual tubules or isolated atrophied tubules; 3, extensive (>50 %) inflammation with large foci of tubular atrophy; 4, nearly all of the interstitium inflamed plus extensive tubular atrophy or necrosis
- *Vascular lesions*: 1, mild thickening of vessel wall, mild infiltration of leukocytes around vessels; 2, moderate thickening of the vessel wall, inflammation of main arteries, small foci of inflammatory cells around interlobular arteries; 3, severe thickening (onion skin pattern) of vessel walls, moderate leukocyte infiltration of small arterial branches; 4, vasculitis, fibrinoid necrosis

The nephritis score is the sum of the individual scores ranging from 0 to 12. The images were acquired using an Axio Imager Z1 microscope (Carl Zeiss MicroImaging, Jena, Germany). All evaluations were performed in a blinded manner.

### Preparation of small mesenteric arteries and functional experiments

A second-order branch of the mesenteric arterial tree was dissected and mounted on two-glass microcannulae in a pressurized myograph (model 110P; Danish Myo Technology, Aarhus, Denmark) as previously described [20]. Media and lumen dimensions were measured with the intraluminal pressure maintained at 45 mmHg.

Endothelium-dependent and -independent relaxations were assessed by measuring the dilatory responses of mesenteric arteries to cumulative concentrations of acetylcholine (ACh, 0.001–100 μM; Sigma-Aldrich, St. Louis, MO, USA) and sodium nitroprusside (SNP, 0.01–100 μΜ; Sigma-Aldrich). Vessels were precontracted with norepinephrine (10 μM; Sigma-Aldrich). This concentration was determined in dose titration experiments to establish the threshold concentration able to elicit similar contractions among the experimental groups. To evaluate NO availability and ROS production, ACh concentration–response curves were constructed before and after 30-minute preincubation with the NOS inhibitor *N*^ω^-nitro-l-arginine methyl ester (l-NAME, 100 μM; Sigma-Aldrich) or the antioxidant ascorbic acid (10 mM, 30-minute preincubation; Sigma-Aldrich). To evaluate whether oxidative stress could influence NO availability, an additional ACh curve was constructed for simultaneous incubation with l-NAME and ascorbic acid.

#### Involvement of NAD(P)H oxidase in endothelium-dependent relaxation

To ascertain the contribution of NAD(P)H oxidase to ED in additional experiments on NZ vessels (n = 6 each group), an ACh curve was constructed after 30-minute incubation with two different NAD(P)H oxidase inhibitors, apocynin (Apo, 10 μM; Fluka-Sigma-Aldrich, St. Gallen, Switzerland) and diphenyleneiodinium (DPI, 10 μM; Sigma-Aldrich) [21]. To assess the possibility that NAD(P)H oxidase could affect NO availability, Ach was infused under simultaneous incubation with l-NAME and Apo or DPI.

#### Detection of vascular superoxide anion generation

The in situ production of superoxide anion was measured by means of the fluorescent dye dihydroethidium (DHE; Sigma-Aldrich) as previously described [22]. Each segment was analyzed simultaneously after incubation with Apo (100 μM) or Krebs solution. Additional incubations with the superoxide scavenger polyethylene glycol superoxide dismutase (PEG-SOD, 600 U ml^−1^; Sigma-Aldrich) were performed as technical positive controls.

### Data analysis

Maximal ACh- and SNP-induced responses (E_max_) were calculated as maximal percentage increments of lumen diameter. The results are presented as mean ± SEM. The statistical significance of relaxation responses was assessed, taking into consideration the time course and treatment by two-way analysis of variance (ANOVA). Other comparisons were made by repeated-measures ANOVA or by one-way ANOVA followed by a Student-Newman-Keuls test, where appropriate. A value of *P* < 0.05 was considered statistically significant. The variable *n* indicates the number of experiments. All analyses were performed using Prism version 5.0 software (GraphPad Software, La Jolla, CA, USA).

## Results

### Renal parameters

#### Proteinuria

In the NZ group, the baseline (weeks 8–12) levels of 24-h urinary protein excretion was 0.154 ± 0.53 mg. It started to increase at 18 weeks (mean 3.5 ± 2.7 mg; *P* = 0.04), and 40 % of animals had proteinuria ≥100 mg/dl. Proteinuria remained stable during the following weeks (4.06 ± 2.8 mg at 24 weeks, 4.9 ± 2.2 mg at 30 weeks; *P* = ns) and increased at 36 weeks (12.5 ± 3 mg; *P* > 0.001) (Table 1).

In the NZ-HCQ group, proteinuria was stable across 12–30 weeks of age, with no significant differences between baseline (0.22 ± 0.2 mg) and 30 weeks (0.35 ± 0.34 mg; *P* = ns); however, significantly higher levels of proteinuria were observed at 36 weeks (mean 12 ± 18 mg; *P* < 0.001) (Table 1).

In control mice, proteinuria was stable across 12–40 weeks of age (mean 1.44 ± 4.1 mg) (Table 1).

#### Anti-dsDNA autoantibody titers

**Fig. 1:**
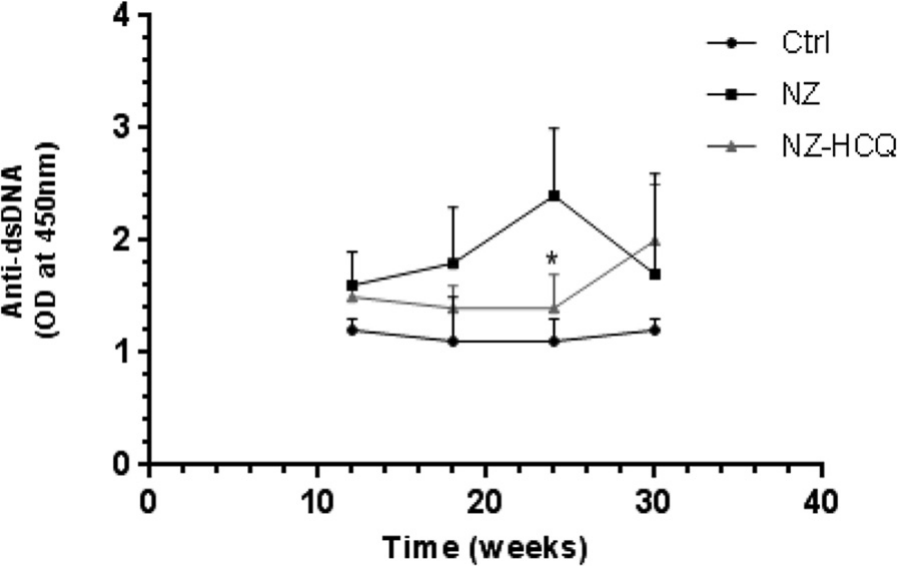
Anti–double-stranded DNA (anti-dsDNA) levels at different time points. The results are expressed as optical density (OD) (mean of the OD readings of serum at 450 nm) as measured using a microtiter plate reader (Ultrospec 2000; Amersham Pharmacia Biotech, Little Chalfont, UK). *P = 0.02 in NZB/W F1 mice treated with hydroxychloroquine (NZ-HCQ) vs. NZB/W F1 (NZ) mice at 24 weeks

Anti-dsDNA antibodies were detectable starting at 12 weeks of age in NZ mice (33 % positivity); however, a significant increase in titers was observed thereafter, with higher serological activity (87 % positivity) at 24 weeks of age (47 % positivity at 18 weeks, 67 % at 30 weeks). In the NZ-HCQ group, anti-dsDNA antibody positivity at 24 weeks of age was 20 %, significantly lower than that in the NZ mice (*P* = 0.002). At 30 weeks of age, the anti-dsDNA positivity was 66 %, with no differences from age-matched, untreated mice. Figure 1 shows the anti-dsDNA titers at different time points in NZ, NZ-HCQ, and Ctrl mice.

#### Nephritis score

Initial histopathological lesions suggestive of nephritis appeared on kidneys from NZ mice killed at 18 weeks of age, then, the degree of nephritis increased progressively until the last observation at age 36 weeks (mean score 2.75 ± 1.25). Similar nephritis scores were observed in kidneys from NZ-HCQ mice at 24, 30, and 36 weeks of age. These data are reported in Table 2.

**Table 2:** Histopathology

Age, wk	Nephritis score (mean ± SD)		*P* value
	NZ	NZ-HCQ	
12	0 ± 0	–	n.s.
18	0.4 ± 0.5	–	n.s.
24	0.75 ± 0.5	2 ± 0.5	n.s.
30	1 ± 0.9	2 ± 0.5	n.s.
36	2.8 ± 1.2	3 ± 4.6	n.s.

*n.s.* not significant, *NZ* NZB/W F1 mice, *NZ-HCQ* NZB/W F1 mice treated with hydroxychloroquine, *SD* standard deviation

No histopathological lesions suggestive of nephritis were detected in control mice.

### Endothelium-dependent relaxation in Ctrl and SLE mice: role of NO availability and ROS production

**Fig. 2:**
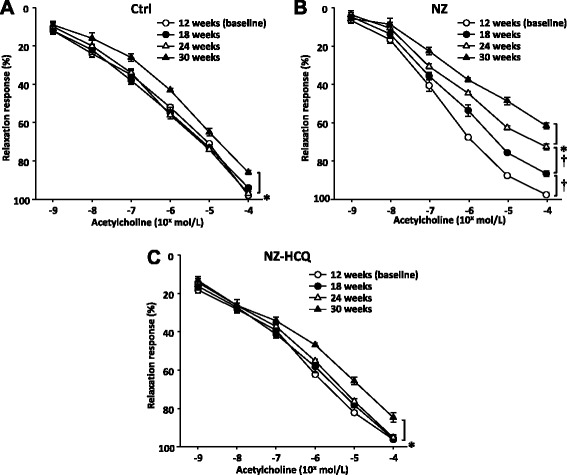
Relaxations in response to acetylcholine in mesenteric arteries at baseline and different time points in control mice (**a**), NZB/W F1 mice (NZ) (**b**) or NZB/W F1 mice treated with hydroxychloroquine (NZ-HCQ) (**c**). Each point represents the mean ± SEM of eight experiments. *P < 0.05; ^†^ *P* < 0.01

**Fig. 3:**
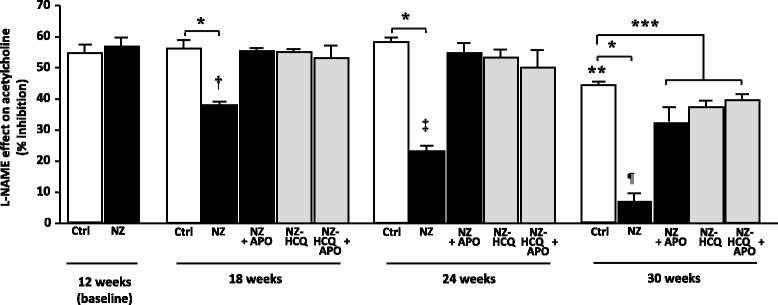
Inhibitory effects of *N* ^ω^-nitro-l-arginine methyl ester (l-NAME) on acetylcholine-induced maximal relaxation at baseline and different time points in control C57BL/6 J mice (Ctrl), NZB/W F1 mice (NZ), and NZB/W F1 mice treated with hydroxychloroquine (NZ-HCQ). Each column represents the mean ± SEM of eight experiments. *P < 0.001; **P < 0.05 vs. other Ctrl groups; ***P < 0.01; ^†^ *P* < 0.01 vs. NZ baseline; ^‡^ *P* < 0.01 vs. 18-week-old NZ; ^¶^ *P* < 0.01 vs. 24-week-old NZ. *Apo* apocynin

In Ctrl animals, relaxation in response to ACh was preserved up to week 24 (E_max_ ACh, 12 weeks: 99.7 ± 0.6 %; 18 weeks: 99.8 ± 0.4 %; 24 weeks: 99.6 ± 0.7 %), but it was attenuated at week 30 (90.0 ± 0.9 %) (Fig. 2a). l-NAME significantly blunted the relaxation in response to ACh at baseline and until 30 weeks, when a reduced inhibitory effect emerged (Fig. 3). Moreover, ascorbic acid was ineffective in changing the vascular response to ACh at baseline (E_max_ ACh + ascorbic acid: 99.1 ± 0.4 %) and until 30 weeks (E_max_ ACh + ascorbic acid, 12 weeks: 98.2 ± 0.7 %; 24 weeks: 98.4 ± 0.9 %; 30 weeks: 98.3 ± 0.6 %), when it normalized the relaxation response to ACh (E_max_ ACh + ascorbic acid: 96.9 ± 0.4 %) and restored the inhibitory effect of l-NAME on ACh (54.8 ± 0.9 %).

In NZ mice, relaxation in response to ACh, though preserved at baseline (12 weeks: 99.8 ± 0.4 %), was significantly reduced at week 18 (87.9 ± 1.1 %; *P* < 0.05 vs. Ctrl), with further progressive decline by week 24 (72.3 ± 1.2 %; *P* < 0.01 vs. Ctrl) and week 30 (62.0 ± 0.6 %; *P* < 0.01 vs. Ctrl) (Fig. 2b). The inhibition of l-NAME on ACh was preserved at baseline (Fig. 3) but started to decline at 18 weeks, with a progressive decrement at 24 and 30 weeks, at which point the response to ACh was virtually resistant to l-NAME (Fig. 2). Ascorbic acid normalized the relaxation in response to ACh in all groups (E_max_ Ach + ascorbic acid, 18 weeks: 94.0 ± 1.2 %; 24 weeks: 93.5 ± 1.2 %; 30 weeks: 95.3 ± 0.6 %; *P* = N.S. vs. baseline) and restored the inhibitory effect of l-NAME on ACh (18 weeks: 52.7 ± 2.4 %; 24 weeks: 50.9 ± 2.1 %; 30 weeks: 53.6 ± 2.9 %). Relaxation in response to SNP started to decline at week 30 among Ctrl mice (decrement above baseline −10.3 ± 1.1 %; *P* < 0.05). A significant decrement of vascular response to SNP at week 30 was also evident in the SLE group (decrement above baseline −24.3 ± 1.4 %; *P* < 0.001), with a greater reduction (*P* < 0.05) compared with the age-matched Ctrl group.

### Effects of HCQ on vascular reactivity

Among NZ-HCQ mice, in the 18-week and 24-week subgroups, HCQ administration normalized the relaxation in response to ACh (Fig. 2c) and restored the inhibition by l-NAME on ACh (Fig. 3). In older NZ animals, HCQ potentiated, without normalizing, the relaxation in response to ACh and partly restored the l-NAME effect on endothelium-dependent dilation (Figs. 2c and 3). HCQ was totally ineffective in modulating endothelial function in Ctrl animals (Additional file 1).

### Involvement of NAD(P)H oxidase in endothelium-dependent relaxation

In the Ctrl group, the relaxation in response to ACh was not modified by Apo at any time point (Additional file 1). In contrast, in the NZ group, Apo, while ineffective at baseline, normalized the response to ACh at 18 and 24 weeks (Additional file 2) and restored the inhibitory effect of l-NAME on ACh (Fig. 3). Of note, at 30 weeks, Apo improved the relaxation in response to ACh (Additional file 2), although it did not normalize it, and partly ameliorated the inhibitory effect of l-NAME on the endothelial agonist (Fig. 3). Similar results were obtained with DPI, which normalized the vascular response to ACh in NZ mice at 18 and 24 weeks and restored the inhibitory effect of l-NAME on ACh (Additional file 3). At 30 weeks, DPI only in part potentiated the response to ACh and the inhibition by l-NAME (Additional file 3).

In the NZ-HCQ groups, Apo incubation was devoid of any effect, at any time, either on the response to ACh or on the inhibition by l-NAME (Fig. 3).

### DHE analysis of vascular superoxide anion generation

**Fig. 4:**
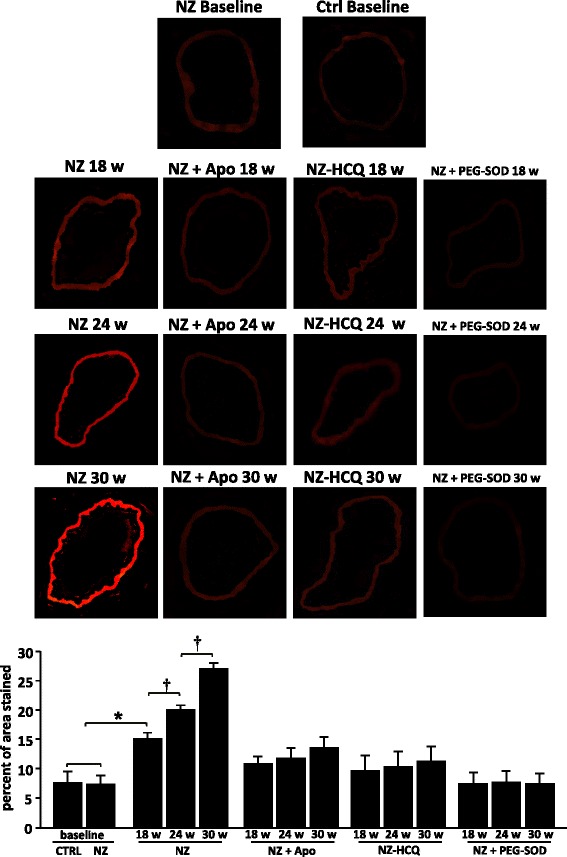
Dihydroethidium (DHE) staining for detection of superoxide production. Representative DHE staining (*upper panels*) and quantitative analysis of the red signal (*lower panels*; original magnification, ×40) in mesenteric arteries from C57BL/6 J mice control mice (Ctrl) and NZB/W F1 mice (NZ) at baseline, from NZ groups without or with apocynin (Apo) or polyethylene glycol superoxide dismutase (PEG-SOD), and from NZB/W F1 mice treated with hydroxychloroquine (NZ-HCQ) at different time points. Each column represents the mean ± SEM of six experiments. *P < 0.001; ^†^ *P* < 0.05

At baseline, DHE red fluorescence revealed a weak and similar mesenteric superoxide anion production among the Ctrl and NZ groups (Fig. 4). In the Ctrl group, advancing age failed to elicit any significant increment of superoxide generation. In contrast, in NZ mice, a dramatic increment of superoxide anion production was already present in the 18-week group, with a progressive production at week 24 and a further generation at 30 weeks (Fig. 4). Incubation with Apo prevented the superoxide production at any time point (Fig. 4). Likewise, in the NZ-HCQ group, superoxide production was completely abrogated (Fig. 4). As technical positive controls, in vessels from NZ mice incubated with PEG-SOD, the superoxide generation was completely abolished (Fig. 4).

## Discussion

In the present study, we have shown that the early administration of HCQ is able to prevent the development of ED in a murine model of SLE.

Endothelial function impairment has been described previously in mice as well as in human SLE [23–30]. In our mouse model, ED occurred at a very early stage of the disease in the absence of overt disease activity. As early as 18 weeks of age, mesenteric small arteries from the NZ group already showed a blunted endothelium-dependent relaxation, together with a reduced inhibitory effect of l-NAME on ACh, and at this stage anti-dsDNA antibodies were positive, and only initial urinary abnormalities were detectable in some mice. DHE analysis revealed a dramatic increment of vascular superoxide anion generation.

To exclude that these effects might be caused by ageing per se, a well-recognized major contributor to vascular functional changes [31], the results from each time of exposure to the disease were compared with data obtained in age-related control groups. Indeed, vessels from control mice showed a preserved inhibitory effect of l-NAME on endothelium-dependent relaxation and no evidence of ROS generation up to 30 weeks. At this age, the control group displayed a reduced NO availability, but this reduction was less pronounced than in the NZ group, without any increased ROS production. Therefore, these data indicate that SLE-associated ED is a functional vascular alteration occurring early in the disease course, regardless of disease activity and aging. These observations fully support the observation made in patients with SLE who might experience premature atherosclerosis independently of traditional risk factors for CV disease [3, 32].

In our model, early and long-term HCQ administration, though ineffective in modulating endothelial function in control mice, normalized the endothelium-dependent relaxation and the inhibitory effect of l-NAME on ACh as well, in both 8-week-old and 24-week-old NZ mice. This protective effect, although still evident, was only partially effective in NZ at 30 weeks of age.

It is well known that renal involvement itself can cause ED. Thus, the observed beneficial effect of HCQ on endothelial function might be consequent to an improvement of kidney disease [33]. However, in our experimental model, this is an unlikely hypothesis, considering that the reversal of ED induced by HCQ occurred before the occurrence of overt kidney disease. In addition, even if chronic administration of HCQ has a positive effect on proteinuria and anti-dsDNA autoantibodies, histological analysis revealed a comparable activity and damage scores in the kidneys from HCQ-treated and untreated mice. In conjunction, these findings indicate that the protective vascular effects by HCQ were not exclusively related to kidney disease and suggest the ability of the drug to interfere with the vascular inflammatory process and the immunological mechanisms accounting for ED, as previously demonstrated [34–37].

Apo, an NAD(P)H oxidase inhibitor, normalized the inhibitory effect of l-NAME on ACh-induced relaxation up to 30 weeks of age. Moreover, DHE staining showed that Apo prevented the SLE-induced vascular ROS at any time point of the disease.

On the basis of these findings, it is conceivable that NAD(P)H oxidase is a major enzymatic pathway that mediates vascular ROS production in this model of SLE, a process that likely initiates at a very early stage of the disease.

Indeed, the present data extend the previous demonstration of the beneficial effect of HCQ on large arteries [17] to the peripheral microcirculation and demonstrate a property of this compound of delaying the onset of vascular functional alterations for a long period.

The main novelty of the data relies on the fact that our work offers a different perspective, given that early disease is the main focus of the study.

Indeed, in 2010, Thacker et al. [23] described an impaired endothelial-dependent vasorelaxation at the aortic level at 36 weeks of age in the same model, in concomitance with an overt disease expression. However, in line with our observations, they also showed an earlier impairment in phenotype and function of endothelial progenitor cells, before full disease expression [23].

More recently, Gómez-Guzmán et al. [17], in the same SLE model, described the protective effect of a short course of HCQ on vascular parameters (hypertension, heart rate, cardiac hypertrophy) as well as on disease-related variables. Similar to us, they found, even if in a large vessel bed such as the aorta, a beneficial effect of HCQ on endothelium-dependent vasodilation on vascular ROS levels and NADPH oxidase activity. However, with respect to our study, the authors started the treatment in an advanced disease stage, after a long-standing autoimmune-mediated inflammatory process. Thus, we can postulate that what we observed is not only the result of an immunological process but also a mixture of metabolism, purely CV detriment, and aging. This is supported by the fact that the administration of a mixture of antioxidants yielded similar results [17].

Some limitations of this study have to be acknowledged. First, we have histological data of the kidney from advanced disease, but no earlier evaluations (18–20 weeks of age) were available. This aspect can account for some discrepancy observed between the proteinuria trend and the kidney histology between treated and untreated mice. Indeed, whereas a delay in proteinuria occurrence has been observed in mice during HCQ treatment, this effect was not reflected by the later histology scores. Second, no serial data on anti-dsDNA antibody activity were available from each mouse, because blood samples were taken only at the time they were killed. This could reduce the accuracy in defining disease progression. Moreover, the mice were given the HCQ in their drinking water; although this is a common practice, variations in individual mice as to intake cannot be excluded.

## Conclusions

This study shows that early treatment with HCQ is able to reduce early ED related to the inflammatory process in a mouse model of SLE. These data may support the clinical observations of the potential beneficial effect of HCQ in preventing vascular damage in human SLE and may support the early introduction of HCQ in patients with recent disease onset.

## Additional files

Additional file 1:
**Supplemental methods and results.** Methods: preparation, mounting and measurements in small arteries and detection of vascular superoxide anion generation. Results: effects of HCQ on vascular reactivity in Ctrl animals. (DOC 31 kb) Additional file 2:
**Relaxation associated with acetylcholine in basal conditions (saline) or in the presence of apocynin (Apo) in mesenteric arteries from NZ animals at baseline and at different time points.** Each point represents the mean of six experiments ± SEM. **P* < 0.05. (PPT 198 kb) Additional file 3:
**Relaxation associated with acetylcholine in basal conditions (saline) or in the presence of** **l** **-NAME, diphenyleneiodinium (DPI) or both in mesenteric arteries from NZ animals at baseline and at different time points.** Each point represents the mean of six experiments ± SEM. **P* < 0.001; ^†^ *P* < 0.01; ^‡^ *P* < 0.05. (PPT 246 kb)

## Abbreviations

ACh: acetylcholine
Apo: apocynin
ANOVA: analysis of variance
Ctrl: C57BL/6 J mice
CV: cardiovascular
DHE: dihydroethidium
DPI: diphenyleneiodinium
dsDNA: double-stranded DNA
ED: endothelial dysfunction
E_max_: maximal acetylcholine- and sodium nitroprusside–induced response
H&E: hematoxylin and eosin
HCQ: hydroxychloroquine
l-NAME: *N*^ω^-nitro-l-arginine methyl ester
NAD(P)H: nicotinamide adenine dinucleotide phosphate
NO: nitric oxide
NZ: NZB/W F1 mice
NZ-HCQ: NZB/W F1 mice treated with hydroxychloroquine
OD: optical density
PAS: periodic acid–Schiff
PEG-SOD: polyethylene glycol superoxide dismutase
ROS: reactive oxygen species
SD: standard deviation
SLE: systemic lupus erythematosus
SNP: sodium nitroprusside
