# Supplementary material for: Early treatment with hydroxychloroquine prevents the development of endothelial dysfunction in a murine model of systemic lupus erythematosus
Source: Arthritis Res Ther. 2015 Oct 6;17:277. doi: 10.1186/s13075-015-0790-3 (PMC4594997; doi:10.1186/s13075-015-0790-3)
Supplement: Additional file 1: — Supplemental methods and results. Methods: preparation, mounting and measurements in small arteries and detection of vascular superoxide anion generation. Results: effects of HCQ on vascular reactivity in Ctrl animals. (DOC 31 kb) [file 13075_2015_790_MOESM1_ESM.doc]

**Additional file 1**

**Methods**

## *Preparation, Mounting and Measurements in Small Arteries*

## Small arteries (150 to 300 µm) were isolated immediately after the animal sacrifice and mounted on a pressurized myograph (110P Danish Myo Technology, Aarhus, Denmark), Vessel segments (2 mm long) were mounted onto 2 glass cannulas, one of which was positioned until the vessel walls were parallel, and equilibrated in physiologic salt solution (mmol/L: NaCl 120, NaHCO_3_ 25, KCl 4.7, KH_2_PO_4_ 1.18, MgSO_4_ 1.18, CaCl_2_ 2.5, EDTA 0.026, and glucose 5.5) bubbled continuously with 95% air and 5% CO_2_ to achieve pH 7.4 at 37°C. Vessels were pressurized at 45 mmHg. All functional experiments were performed by measuring dilatory responses to agonists in vessels precontracted with norepinephrine (Sigma-Aldrich, Saint Louis, Missouri, USA).

***Detection of vascular superoxide anion generation.***

The *in situ* production of superoxide anion was measured by means of the fluorescent dye dihydroethidium (DHE, Sigma-Aldrich, Saint Louis, Missouri, USA ). Three slides per segment were analyzed simultaneously after incubation with antagonists or Krebs solution (Sigma-Aldrich, Saint Louis, Missouri, USA) at 37°C for 30 min. Krebs-HEPES buffer containing 2 μM DHE was then applied onto each section and evaluated under fluorescence microscopy. The percentage of arterial wall area stained with the red signal was evaluated using an imaging software (McBiophotonics Image J; National Institutes of Health, Bethesda, Maryland, USA).

**Results**

***Effects of HCQ on Vascular Reactivity in Ctrl animals***

In Ctrl animals, HCQ was totally ineffective in modulating endothelial function at 12 weeks (E_max_ ACh: 97.0±0.9%; ACh + L-NAME: 44.0±1.2%; inhibition: 53±1.2%), 18 weeks (E_max_ ACh: 95.1±0.8%; ACh + L-NAME: 43.3±1.3%; inhibition: 51.7±1.0%) or at 30 weeks (E_max_ ACh: 86.3±0.6%; ACh + L-NAME: 50.0±1.1%; inhibition: 36±1.5%).
