# Supplementary material for: Early treatment with hydroxychloroquine prevents the development of endothelial dysfunction in a murine model of systemic lupus erythematosus
Source: Arthritis Res Ther. 2015 Oct 6;17:277. doi: 10.1186/s13075-015-0790-3 (PMC4594997; doi:10.1186/s13075-015-0790-3)
Supplement: Additional file 2: — Relaxation associated with acetylcholine in basal conditions (saline) or in the presence of apocynin (Apo) in mesenteric arteries from NZ animals at baseline and at different time points. Each point represents the mean of six experiments ± SEM. *P < 0.05. (PPT 198 kb) [file 13075_2015_790_MOESM2_ESM.ppt]

## Slide 1
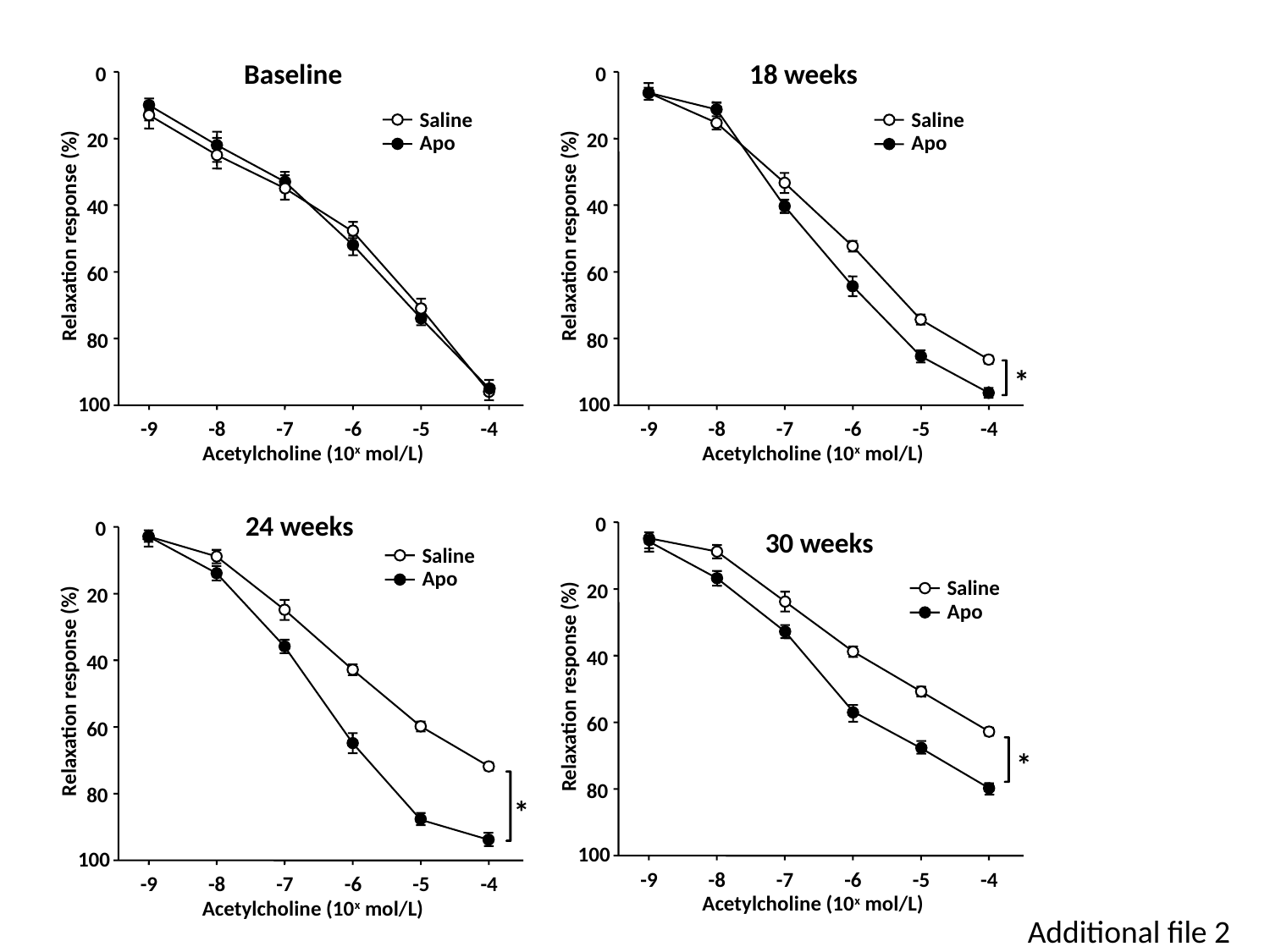

Baseline
18 weeks
0
0
Saline
Saline
20
20
Apo
Apo
40
40
Relaxation response (%)
Relaxation response (%)
60
60
80
80
*
100
100
-9
-8
-7
-6
-5
-4
-9
-8
-7
-6
-5
-4
Acetylcholine (10x mol/L)
Acetylcholine (10x mol/L)
24 weeks
0
0
30 weeks
Saline
Apo
Saline
20
20
Apo
40
40
Relaxation response (%)
Relaxation response (%)
60
60
*
80
80
*
100
100
-9
-8
-7
-6
-5
-4
-9
-8
-7
-6
-5
-4
Acetylcholine (10x mol/L)
Acetylcholine (10x mol/L)
Additional file 2
