# Supplementary material for: Early treatment with hydroxychloroquine prevents the development of endothelial dysfunction in a murine model of systemic lupus erythematosus
Source: Arthritis Res Ther. 2015 Oct 6;17:277. doi: 10.1186/s13075-015-0790-3 (PMC4594997; doi:10.1186/s13075-015-0790-3)
Supplement: Additional file 3: — Relaxation associated with acetylcholine in basal conditions (saline) or in the presence of l -NAME, diphenyleneiodinium (DPI) or both in mesenteric arteries from NZ animals at baseline and at different time points. Each point represents the mean of six experiments ± SEM. *P < 0.001; † P < 0.01; ‡ P < 0.05. (PPT 246 kb) [file 13075_2015_790_MOESM3_ESM.ppt]

## Slide 1
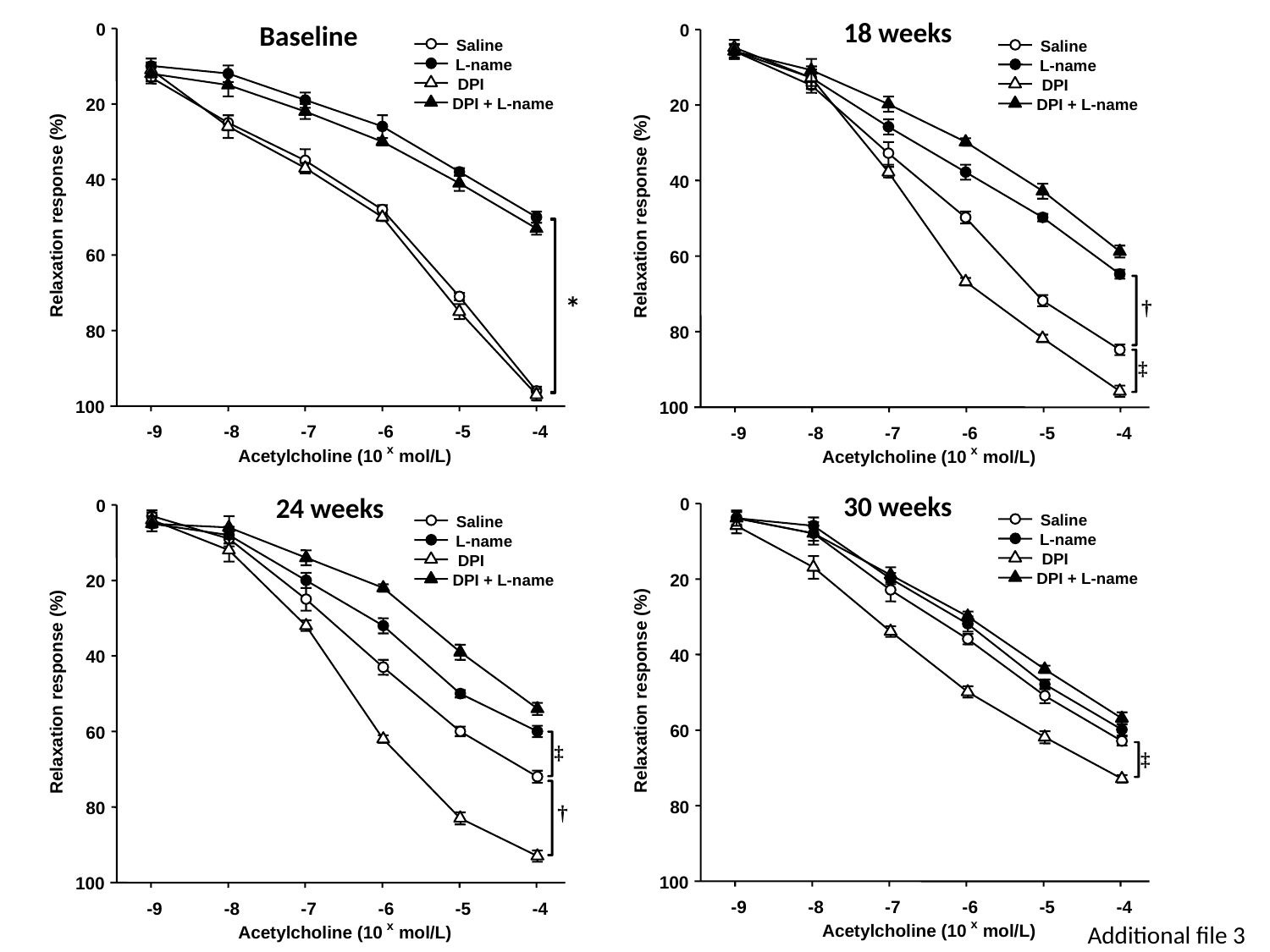

18 weeks
0
Saline
L-name
DPI
DPI + L-name
20
40
Relaxation response (%)
60
†
80
100
-9
-8
-7
-6
-5
-4
x
Acetylcholine (10
 mol/L)
Baseline
0
Saline
L-name
DPI
DPI + L-name
20
40
Relaxation response (%)
60
*
80
100
-9
-8
-7
-6
-5
-4
x
Acetylcholine (10
 mol/L)
‡
30 weeks
24 weeks
0
0
Saline
Saline
L-name
L-name
DPI
DPI
DPI + L-name
20
DPI + L-name
20
40
40
Relaxation response (%)
Relaxation response (%)
60
60
‡
‡
†
80
80
100
100
-9
-8
-7
-6
-5
-4
-9
-8
-7
-6
-5
-4
x
x
Acetylcholine (10
 mol/L)
Acetylcholine (10
 mol/L)
Additional file 3
